# Supplementary material for: Splice-Junction-Based Mapping of Alternative Isoforms in the Human Proteome
Source: Cell Rep. Author manuscript; Available in PMC 2020 Jan 15. (PMC6961840; doi:10.1016/j.celrep.2019.11.026)
Supplement: 2 [file NIHMS1546469-supplement-2.zip › DF1/PXD010154/Heart/Heart_16_TTN_RVEAEPAEEVTIMEEK.pdf]

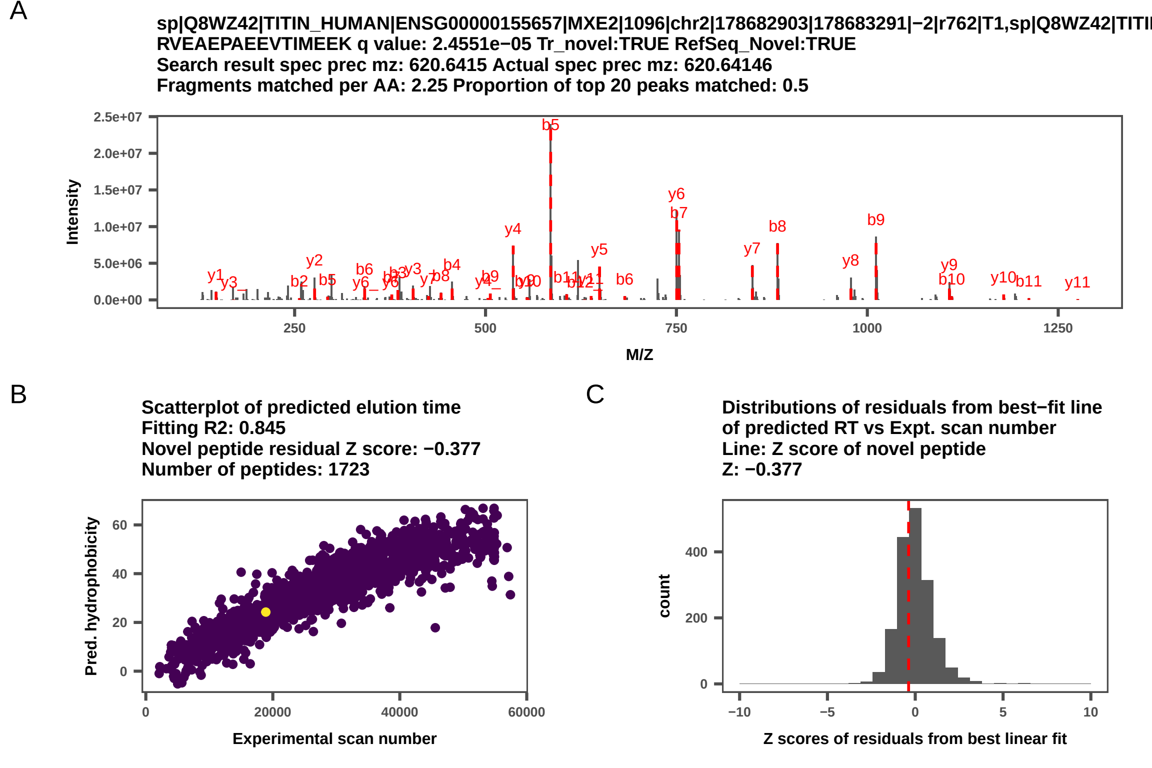
A
sp|Q8WZ42|TITIN_HUMAN|ENSG00000155657|MXE2|1096|chr2|178682903|178683291|−2|r762|T1,sp|Q8WZ42|TITI
RVEAEPAEEVTIMEEK q value: 2.4551e−05 Tr_novel:TRUE RefSeq_Novel:TRUE
Search result spec prec mz: 620.6415 Actual spec prec mz: 620.64146
Fragments matched per AA: 2.25 Proportion of top 20 peaks matched: 0.5
Intensity
2.5e+07
2.0e+07
1.5e+07
1.0e+07
5.0e+06
0.0e+00
b5
y6
b7
b8
b9
y4
y5
y7
y8
y9
b10
y10
b11
y11
y1
y2
b2
b6
b4
M/Z
250
500
750
1000
1250
B
Scatterplot of predicted elution time
Fitting R2: 0.845
Novel peptide residual Z score: −0.377
Number of peptides: 1723
Pred. hydrophobicity
60
40
20
0
0
20000
40000
60000
Experimental scan number
C
Distributions of residuals from best−fit line
of predicted RT vs Expt. scan number
Line: Z score of novel peptide
Z: −0.377
count
400
200
0
−10
−5
0
5
10
Z scores of residuals from best linear fit
